# Impact of Contour Boundary Offsets on 4D Flow CMR-Derived Intracardiac Haemodynamic Parameters

**DOI:** 10.3390/jcdd13060280

**Published:** 2026-06-22

**Authors:** Alexander Gall, Rui Li, Ciaran Grafton-Clarke, Zia Mehmood, Kurian Thampi, Amanda Noyes, David Hewson, Victoria Underwood, Rebekah Girling, David Marlevi, Peter P Swoboda, Rob J. van der Geest, Gareth Matthews, Pankaj Garg

**Affiliations:** 1 Norwich Medical School, University of East Anglia, Norwich NR4 7TJ, UK; 2 Cardiology Department, Norfolk and Norwich University Teaching Hospitals, Norwich NR4 7UY, UK; 3 Department of Molecular Medicine and Surgery, Karolinska Institute, SE-171 76 Solna, Sweden; 4 Leeds Institute of Cardiovascular and Metabolic Medicine, University of Leeds, Leeds LS2 9JT, UK; 5 Leiden University Medical Centre, 2333 Leiden, The Netherlands

**Keywords:** 4D flow, cardiovascular magnetic resonance, haemodynamics, kinetic energy, vorticity, viscous energy loss

## Abstract

Four-dimensional (4D) flow cardiovascular magnetic resonance assesses advanced haemodynamic parameters like kinetic energy (KE), vorticity, and viscous energy loss (vEL). However, gradient-based metrics (vorticity, vEL) are highly sensitive to partial volume effects near the fluid–tissue boundary. This study investigated the impact of systematic contour boundary offsets on these parameters to standardise analysis. Five cases underwent 4D flow imaging. Deep learning-derived automated segmentations of the cardiac chambers were generated. Haemodynamics were analysed using three contouring methods: the baseline mask, a one-voxel inward offset, and a two-voxel inward offset. KE, vorticity, and vEL decreased progressively with larger offsets. KE declined modestly with erosion (by approximately 18% and 35% at one- and two-voxel offsets, respectively), a reduction commensurate with the loss of integration volume rather than the removal of boundary artefacts. By contrast, the gradient-based metrics were disproportionately sensitive to boundary proximity. In the left ventricle, mean full-cycle vorticity decreased from 249.6 ± 79.9 s^−1^ (baseline) to 157.0 ± 60.4 s^−1^ (two-voxel offset; Hedges’ g 2.11), whilst vEL decreased from 549.4 ± 303.0 µW to 351.3 ± 230.0 µW (Hedges’ g 2.00). A one-voxel inward offset optimally reduces boundary noise for sensitive gradient-based parameters. While KE analysis remains satisfactory using unmodified baseline contours, we recommend the uniform application of a one-voxel offset across all parameters to ensure methodological simplicity and pipeline standardisation.

## 1. Introduction

Four-dimensional (4D) flow cardiovascular magnetic resonance (CMR) imaging has emerged as a powerful non-invasive tool for evaluating complex intracardiac and endovascular haemodynamics [1,2]. Beyond standard flow quantification, 4D flow CMR permits the derivation of advanced fluid dynamic parameters, such as kinetic energy (KE), vorticity, and viscous energy loss (vEL), which offer deeper pathophysiological insights into cardiovascular diseases [3,4,5].

Kinetic energy provides a global assessment of the blood’s motion and the work done by the heart to accelerate blood [1,6]. Vorticity characterises the local spinning motion of the blood, providing an index of flow disturbance and vortex formation [4,7]. Viscous energy loss, calculated via the viscous dissipation of kinetic energy, serves as a marker for cardiac workload and the efficiency of blood transport [4,8]. However, the accurate quantification of these parameters relies heavily on precise spatial resolution and accurate region of interest boundary delineation [9,10,11].

A significant challenge in 4D flow CMR analysis is the presence of partial volume effects, phase dispersion, and reduced signal-to-noise ratio at the fluid–tissue interface (endocardial or vessel border) [5,12]. Because vEL and vorticity are mathematically dependent on spatial velocity gradients, which are typically highest near the perimeter of the cardiac chamber (boundary layer), these calculations are particularly susceptible to boundary noise [4,8,9,10]. Including static or slow-moving tissue voxels within the fluid domain can introduce errors into vEL and vorticity calculations [1].

To mitigate these boundary-related inaccuracies, systematic erosion of the segmentation masks can be employed [13,14,15,16]. The purpose of this mechanistic study was to evaluate the effect of contour boundary offsets on the quantification of 4D flow-derived haemodynamic markers. By comparing analyses performed using baseline contours derived from magnitude images with masks subjected to 1-voxel and 2-voxel inward offsets, this study sought to identify an approach that maximises accuracy and reliability in the evaluation of vorticity and vEL.

## 2. Materials and Methods

### 2.1. Study Population

This mechanistic proof-of-concept study included five representative cases identified from the prospective PREFER-CMR registry, which enrols patients undergoing clinically indicated CMR. Demographic, biochemical, and volumetric characteristics available for the study cohort are summarised in Table 1.

### 2.2. MRI Acquisition

CMR studies were performed on a 1.5 Tesla MAGNETOM Sola (Siemens Healthineers, Erlangen, Germany) system equipped with BioMatrix Body 18 channel and 32 channel spine coil technology. The CMR protocol comprised localiser/survey images, cine imaging, late gadolinium enhancement (LGE) imaging, and 4D-flow acquisition. 4D flow was acquired with retrospective ECG gating and reconstructed into 30 cardiac phases. Acquisition was performed during free breathing without respiratory navigator gating using a non-breath-hold protocol, with volumetric coverage of the heart and thoracic aorta acquired in the sagittal plane. 4D flow was acquired following administration of a gadolinium-based contrast agent.

4D flow imaging was performed using a spoiled gradient-echo–based velocity-encoded sequence with a flip angle of 15°. Echo time was approximately 2.7 ms. A segmented acquisition scheme was used, with an underlying gradient-echo repetition time on the order of 4–5 ms. Spatial resolution was approximately isotropic, with in-plane voxel dimensions of ~3.1 × 3.1 mm^2^ and a slice thickness of 3.1 mm, acquired without inter-slice gap. In-plane FOV and phase FOV were adjusted to ensure full coverage of the heart and thoracic aorta, resulting in an effective FOV of approximately 200 × 256 mm^2^, with minor variation according to patient size and protocol variant. Parallel imaging was applied using GRAPPA acceleration in the phase-encoding direction with an acceleration factor of 2 and no slice-direction acceleration. Temporal resolution was heart-rate dependent for the reconstructed 30 cardiac phases. Velocity encoding (VENC) was set to 150 cm/s by default in all five cases. Typical acquisition time for the free-breathing, non-navigator 4D-flow protocol was 8–12 min, depending on heart rate and spatial coverage.

### 2.3. Pre-Processing and Segmentation

Background phase-offset correction and related pre-processing steps were performed offline during post-processing using MASS research software (Version 2025-EXP; Leiden University Medical Center, Leiden, The Netherlands). Quality control included visual inspection for residual phase offsets and velocity aliasing, with additional corrective steps applied as required prior to quantitative flow analysis.

The magnitude images from the 4D flow dataset were reconstructed into an axial stack, with a voxel size of 3 mm × 3 mm × 3 mm. Initial automated segmentation of the cardiac chambers was performed on the 4D flow magnitude images to define the baseline anatomical boundary. These segmentations were time resolved, meaning contours were generated for each of the 30 cardiac phases. This was achieved using a recently developed deep learning model based on a 3D full-resolution nnU-Net architecture [17]. The model was trained on 90 manually segmented whole-heart volumetric phases from 30 subjects and was designed for automated extraction of volumetrics and haemodynamics from 4D flow CMR magnitude images, to include both ventricles, both atria, the thoracic aorta and the main pulmonary artery. Internal validation of this model demonstrated high spatial overlap with expert manual contours, with an overall mean Dice similarity coefficient of 0.88 across all cardiac chambers. All baseline automated masks underwent visual inspection by an experienced observer; no manual adjustments were required for the five cases included in this study.

**Figure 1:**
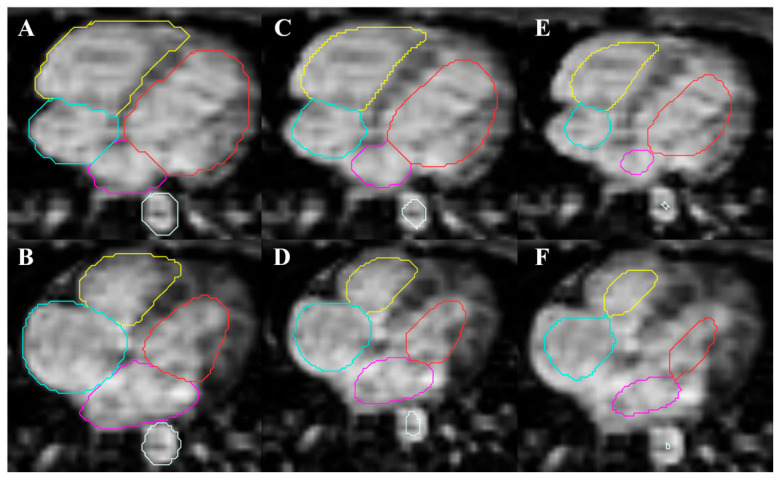
Methodological application of systematic contour boundary offsets. Representative axial 4D flow magnitude images demonstrating the three segmentation mask methods applied across the cardiac chambers and descending aorta. Panels (**A**,**B**) show the baseline automated contours; panels (**C**,**D**) show a 1-voxel inward morphological erosion (3 mm offset); panels (**E**,**F**) show a 2-voxel inward morphological erosion (6 mm offset), displayed at end-diastole (**top row**) and end-systole (**bottom row**). Red = left ventricle. Yellow = right ventricle. Pink = left atrium. Turquoise = right atrium. White = descending aorta.

From this baseline AI-derived segmentation, three distinct analysis masks were generated for each case to evaluate the impact of boundary proximity. The voxel offsets were generated using a standard 2D slice-by-slice morphological erosion operation to ensure a uniform inward boundary shift (Figure 1). Three distinct analysis masks were generated to evaluate boundary proximity: a baseline unmodified mask (Method A), and two subsequent masks subjected to inward morphological erosions of 1-voxel (3 mm; Method B) and 2-voxels (6 mm; Method C), respectively.

### 2.4. Haemodynamic Analysis

Advanced haemodynamic analysis was performed using MASS research software (Version 2025-EXP, Leiden University Medical Center, Leiden, The Netherlands). Kinetic energy was calculated directly from the velocity field and assumed blood density of 1060 kg/m^3^. To determine the sensitivity of this parameter to boundary proximity, kinetic energy was integrated over the segmented volume across the cardiac cycle for the baseline, one-voxel offset, and two-voxel offset contouring methods. Vorticity was calculated as the curl of the velocity vector field and integrated over the segmented volume across the cardiac cycle for each contouring method [18]. Viscous energy loss was calculated from the Navier–Stokes viscous dissipation function by integrating spatial velocity gradients across the three-dimensional fluid domain for each contouring method [19]. All haemodynamic parameters were evaluated both as full cardiac cycle (full RR) integrals and peak systole, peak E wave, and peak A wave.

### 2.5. Qualitative Visual Grading

To evaluate the visual impact of contour boundary offsets on haemodynamic mapping, a qualitative grading system was applied to the vorticity and viscous energy loss heatmaps for each contouring method by a single experienced operator. Interface noise was visually graded on a 4-point scale: 0 = Severe noise obscuring true flow; 1 = Moderate noise; 2 = Mild noise; 3 = Clean interface with minimal to no boundary artifact.

### 2.6. Statistical Analysis

Continuous variables are expressed as mean ± standard deviation. Comparisons between the three contouring methods (baseline, 1-voxel offset, and 2-voxel offset) were performed using the non-parametric Friedman test. Post hoc pairwise comparisons between specific methods were performed using the Wilcoxon signed-rank test. To account for multiple post hoc comparisons, a Bonferroni correction was applied (0.05/3), yielding an adjusted significance threshold of *p* < 0.0167 for pairwise testing. Paired Hedges’ g effect sizes were calculated for the gradient-based metrics (vorticity and viscous energy loss) as the mean of the within-subject paired differences divided by the standard deviation of those differences, incorporating the Hedges’ small-sample correction factor (J = 1–3/(4(n − 1) − 1)). Because kinetic energy is a volume-extensive quantity, for which this paired statistic reflects the consistency rather than the magnitude of a near-proportional volumetric reduction, its sensitivity to boundary erosion is instead summarised as the mean percentage reduction relative to baseline. A two-tailed *p*-value < 0.05 was considered statistically significant for the overall Friedman test. Statistical analyses were performed using Python v3.12.4 (SciPy and Statsmodels libraries).

## 3. Results

### 3.1. Baseline Characteristics

The study cohort comprised five representative cases with a mean age of 76.6 ± 3.4 years, of which 60% were male. The mean heart rate was 72 ± 11 beats per minute, and the mean body surface area was 1.85 ± 0.28 square metres. Baseline volumetric analysis demonstrated a mean left ventricular end-diastolic volume of 176.0 ± 76.5 mL, a left ventricular end-systolic volume of 65.3 ± 27.6 mL, and a mean left ventricular ejection fraction of 62.9 ± 5.5% (Table 1).

### 3.2. Kinetic Energy Response to Contour Boundary Offsets

Across all cardiac chambers, full-cardiac cycle kinetic energy demonstrated a reduction with increasing inward contour offsets. In the left ventricle, mean kinetic energy decreased from 1.86 ± 1.13 mJ using the baseline contour to 1.64 ± 1.04 mJ with a one-voxel offset, and to 1.37 ± 0.94 mJ with a two-voxel offset. A similar decline was observed in the right ventricle, where mean kinetic energy reduced from 1.28 ± 0.57 mJ at baseline to 1.11 ± 0.53 mJ and 0.87 ± 0.44 mJ following one-voxel and two-voxel erosions, respectively. Atrial measurements exhibited a corresponding pattern, with left atrial mean kinetic energy declining from 1.12 ± 0.69 mJ at baseline to 0.87 ± 0.57 mJ with a one-voxel offset, and right atrial kinetic energy decreasing from 1.29 ± 0.45 mJ to 1.05 ± 0.41 mJ for the respective masks. This relative stability is demonstrated by the magnitude of percentage change. While gradient-based metrics exhibited massive relative signal loss upon morphological erosion, kinetic energy demonstrated substantial resistance to boundary modifications, retaining the vast majority of its central signal. Expressed as a fractional reduction relative to baseline, full-cardiac-cycle kinetic energy fell by 13.1%, 14.8%, 23.6% and 19.8% (left ventricle, right ventricle, left atrium and right atrium, respectively) at a one-voxel offset, and by 29.4%, 33.5%, 42.3% and 35.8% at a two-voxel offset.

### 3.3. Impact of Contour Offsets on Vorticity

**Figure 2:**
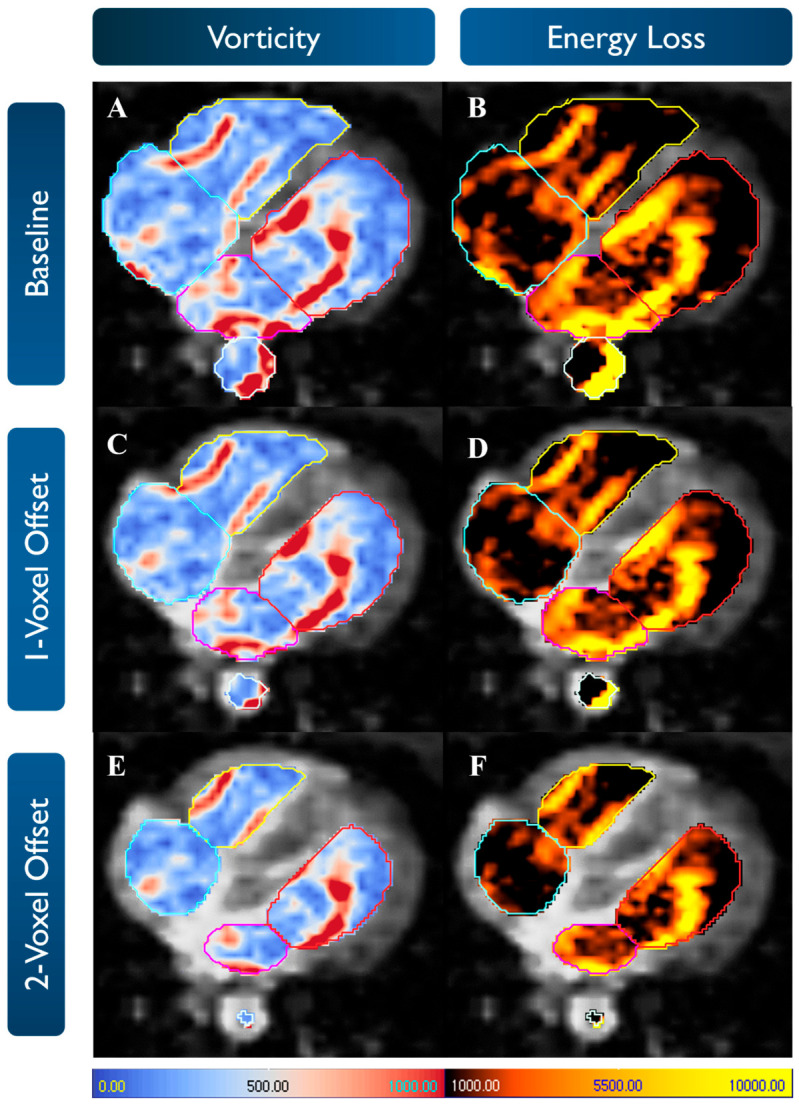
Qualitative impact of boundary erosion on advanced haemodynamic mapping. Representative colour-coded heatmaps of vorticity (**left column**) and viscous energy loss (**right column**) mapped onto the corresponding axial 4D flow magnitude segmentation masks at a single cardiac phase. Panels (**A**,**B**) show baseline masks with high-magnitude signal at the fluid–tissue boundary, particularly evident at the apical lateral left ventricle and left atrium in this slice. Panels (**C**,**D**) show the effect of a 1-voxel offset, with reduced peripheral signal and preservation of the central flow pattern. Panels (**E**,**F**) show the effect of a 2-voxel offset, with further restriction of the analysed flow domain, including almost complete erosion of the ascending aorta mask in this slice. Scale bars: vorticity = 1/s; viscous energy loss = μW. Red = left ventricle. Yellow = right ventricle. Pink = left atrium. Turquoise = right atrium. White = aorta.

**Figure 3:**
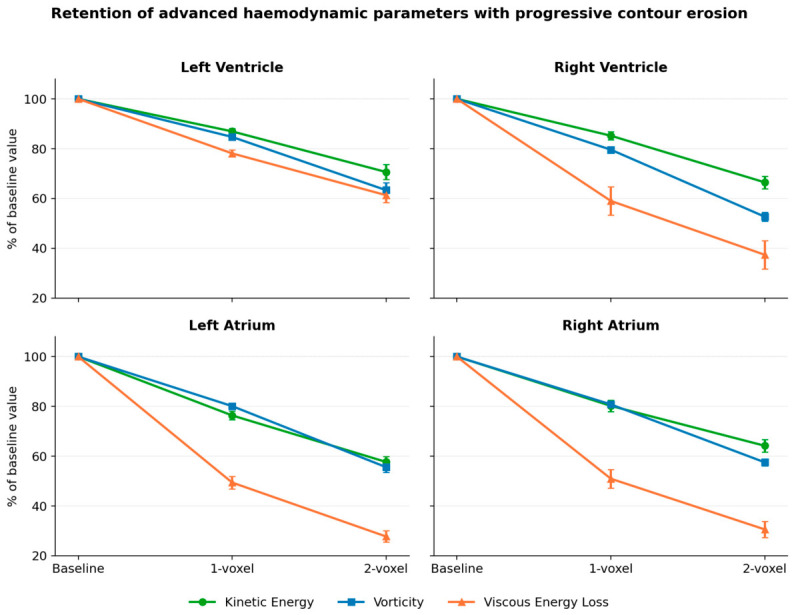
Full-cardiac-cycle kinetic energy, vorticity, and viscous energy loss for each cardiac chamber, expressed as a percentage of the baseline (unmodified-contour) value at one- and two-voxel inward offsets. Points are mean ± standard error of the mean across the five cases.

Across all chambers, vorticity decreased progressively from the baseline mask to the 1-voxel offset and again to the 2-voxel offset (overall Friedman *p* < 0.01 for all chamber-level comparisons). Representative maps are shown in Figure 2, the full-cycle relative changes across all parameters are summarised in Figure 3, with phase-specific trends shown in Supplementary Figure S1.

For full-RR measurements, mean vorticity declined in the left ventricle (LV) from 249.6 ± 79.9 s^−1^ at baseline to 211.1 ± 61.0 s^−1^ with a 1-voxel offset and 157.0 ± 60.4 s^−1^ with a 2-voxel offset. Corresponding reductions were observed in the right ventricle (RV) (196.4 ± 23.3 to 156.6 ± 22.2 to 103.8 ± 16.9 s^−1^), left atrium (LA) (237.6 ± 63.9 to 190.4 ± 52.8 to 132.0 ± 37.9 s^−1^), and right atrium (RA) (243.4 ± 48.7 to 197.3 ± 43.8 to 140.9 ± 35.2 s^−1^) (Table 2). As detailed in Figure 3, the application of a single-voxel erosion resulted in substantial relative reductions in full-cycle vorticity, most notably in the right atrium (19.2%) and right ventricle (20.4%).

The same stepwise pattern was present for peak systolic, peak E-wave, and peak A-wave vorticity in each chamber (Supplementary Figure S1, Table S1).

### 3.4. Impact of Contour Offsets on Viscous Energy Loss

vEL likewise decreased progressively from the baseline mask to the 1-voxel offset and to the 2-voxel offset in all chambers (overall Friedman *p* < 0.01 for all chamber-level comparisons) (Figure 3).

For full-RR measurements, mean vEL declined in the LV from 549.4 ± 303.0 µW at baseline to 434.9 ± 253.0 µW with a 1-voxel offset and 351.3 ± 230.0 µW with a 2-voxel offset. Corresponding reductions were observed in the RV (481.5 ± 173.1 to 279.6 ± 106.1 to 173.6 ± 71.9 µW), LA (828.4 ± 505.2 to 426.9 ± 314.0 to 240.2 ± 180.4 µW), and RA (833.5 ± 223.8 to 429.1 ± 154.4 to 264.0 ± 132.2 µW) (Table 2).

Peak systolic, peak E-wave, and peak A-wave vEL values also declined progressively with each additional inward offset (Supplementary Figure S1, Table S2). The largest relative full-RR reductions were observed for vEL in the RV and atria. Viscous energy loss proved to be the parameter most sensitive to boundary proximity. A single-voxel offset removed approximately half of the measured full-cycle physiological signal in the atria (left atrium: 50.6%; right atrium: 49.1%), and 41.0% in the right ventricle (Figure 3).

### 3.5. Qualitative Impact of Contour Offsets

Qualitative visual grading corroborated the quantitative findings, demonstrating a marked reduction in boundary noise with progressive voxel erosion. At baseline, viscous energy loss maps exhibited near-universal severe interface noise (mean grade 0.2), while baseline vorticity exhibited moderate-to-mild noise (mean grade 1.6). The application of a 1-voxel offset substantially improved map quality, yielding mild-to-clean interfaces for both vorticity (mean grade 2.6) and viscous energy loss (mean grade 2.2). While a 2-voxel offset produced the cleanest maps (mean grade 3.0 for vorticity; 2.4 for energy loss), visual inspection confirmed that this deeper erosion visibly truncated the central physiological flow core. Table S3 details full analysis.

## 4. Discussion

### 4.1. Physiological and Methodological Implications of Voxel Erosion

This mechanistic study evaluated the impact of systematic contour boundary offsets on the quantification of four-dimensional flow-derived advanced haemodynamic parameters. The inclusion of kinetic energy analysis reveals a divergent haemodynamic response to boundary erosion. The gradient-based parameters of vorticity and viscous energy loss exhibited disproportionate sensitivity to fluid–tissue interface proximity, with paired Hedges’ g effect sizes for the sequential erosion steps reaching values of up to approximately 5.0 (Table 2), reflecting large and highly consistent measurement shifts. Kinetic energy also declined with erosion, by approximately 18% at a one-voxel and 35% at a two-voxel offset; however, in contrast to the gradient-based metrics, this reduction is commensurate with the proportion of chamber volume removed rather than with the elimination of boundary artefact.

This divergent sensitivity is intrinsically linked to the mathematical derivation of the respective parameters. Kinetic energy quantifies the absolute magnitude of blood motion within the chamber and is predominantly driven by higher-velocity flow located within the central fluid domain. Near the endocardial border, physiological blood velocity naturally approaches zero due to the no-slip boundary condition. Consequently, the peripheral voxels eroded during contour offsetting represent a relatively minor fraction of the total kinetic energy. Because kinetic energy does not rely upon the spatial derivatives of velocity, it is not susceptible to the mathematical amplification of partial volume effects and phase dispersion that severely confounds gradient-based calculations at the anatomical interface. The measured decline in kinetic energy following morphological erosion is therefore a physiologically expected consequence of reducing the total analysed volume, rather than the successful removal of artefactual noise.

Conversely, the extreme sensitivity observed in the spatial derivative parameters is consistent with the mathematical formulation of viscous energy loss, which depends on the square of the velocity gradients. In four-dimensional flow cardiovascular magnetic resonance, the fluid–tissue interface is a region of marked velocity transition. Consequently, even small amounts of boundary noise or partial-volume contamination can be exponentially amplified in the dissipation calculation. Crucially, physiological viscous dissipation occurs primarily at the fluid–tissue interface due to friction within the boundary layer. However, at a macroscopic spatial resolution of 3 × 3 × 3 mm, this thin physiological boundary layer is inextricably merged with partial-volume artefact. Consequently, any morphological erosion inherently sacrifices true physiological viscous energy loss in the pursuit of noise reduction.

At the acquired spatial resolution, a two-voxel offset represents substantial volumetric erosion. Using the cohort mean left ventricular end-diastolic volume and approximating the chamber as a sphere, a 6 mm shell erosion removes roughly 43 percent of the total volume. This explains why the two-voxel approach produced pronounced reductions in measured left ventricular vorticity and viscous energy loss, reflecting not only reduced boundary-related contamination but also the exclusion of a sizeable proportion of physiological flow. The magnitude of this reduction also varied by chamber geometry, with the larger changes observed in the right ventricle and atria plausibly related to their higher surface-area-to-volume ratios, thinner walls, and greater structural complexity. Furthermore, the reliance on a standard velocity encoding of 150 cm/s inherently reduces the velocity-to-noise ratio within the slow-moving atrial peripheries, disproportionately amplifying background noise in these low-velocity regions and further degrading the reliability of gradient-based measurements near the fluid–tissue interface. In addition, because vorticity and viscous energy loss are derived from spatial velocity gradients, their fidelity is further governed by the reconstructed temporal resolution, which determines how faithfully transient near-wall flow structures are captured across the cardiac cycle. The sensitivity of these gradient-based metrics to erosion is therefore expected to scale with the acquired spatial resolution, and a systematic multi-resolution evaluation (imaging the same subjects at differing voxel sizes) represents an important priority for future work.

### 4.2. Methodological Recommendations for Advanced Haemodynamic Parameters

The findings of this mechanistic study necessitate a parameter-specific understanding of boundary interactions, yet they also highlight the need for a practical approach to four-dimensional flow image analysis. For mathematically derived gradient-based metrics, particularly viscous energy loss and vorticity, uncorrected baseline contours introduce significant boundary noise that renders absolute values clinically unreliable. In these instances, applying a systematic one-voxel inward offset provides an essential standardisation that successfully improves the artefactual interface contamination while preserving the central physiological flow core. The selection of a one-voxel offset reflects a principled minimum-erosion strategy rather than an arbitrary choice. At the acquired resolution (approximately 3.1 mm), the partial-volume contamination shell at the fluid–tissue interface is of the order of a single voxel in depth, such that a one-voxel inward erosion is the minimal offset capable of removing this contaminated layer. By contrast, a two-voxel offset removes approximately 43% of the chamber volume and demonstrably truncates the central physiological flow core; the one-voxel offset therefore represents the principled minimum that clears boundary artefacts without unnecessary loss of physiological signal.

An important caveat concerns the distinction between noise reduction and physiological accuracy. At the present spatial resolution the physiological near-wall boundary layer is effectively inseparable from partial-volume artefact, so any erosion that suppresses interface noise will necessarily also remove a degree of genuine near-wall information. We therefore frame the one-voxel offset as a pragmatic compromise that improves measurement reproducibility and noise rejection, rather than as a claim of improved physiological accuracy, and we caution that erosion-based approaches may attenuate small-scale near-wall vortical structures of physiological or pathological interest [20,21].

Conversely, the decline in kinetic energy under erosion reflects the predictable loss of integration volume rather than the correction of gradient noise; because the eroded near-wall voxels carry relatively little kinetic energy, applying a moderate, standardised erosion does not distort the parameter in the artefactual manner that affects the gradient-based metrics. While calculating kinetic energy using unmodified baseline anatomical contours remains entirely physiologically sound and satisfactory, adopting differing segmentation boundaries for individual haemodynamic parameters within the same dataset introduces logistical complexity. Therefore, to ensure methodological simplicity and to streamline automated analysis pipelines, we recommend the uniform application of a one-voxel inward offset across all advanced haemodynamic parameters. Employing a single, standardised eroded mask ensures the robust quantification of highly sensitive spatial derivatives without materially compromising the consistency of the kinetic energy data, which tolerates this mild, systematic morphological erosion.

### 4.3. Clinical Implications

Advanced four-dimensional flow-derived parameters hold significant promise as early markers of subclinical cardiac dysfunction, offering unique insights into ventricular efficiency and myocardial work. However, the extreme sensitivity of gradient-based metrics to boundary noise dictates that without rigorous standardisation of contouring, the artefactual variation caused by endocardial partial volume effects could lead to the misinterpretation of haemodynamic severity. These advanced flow metrics complement a broader landscape of imaging-driven and computational approaches to regional left ventricular haemodynamic characterisation, including patient-specific computational fluid dynamics modelling derived from phase-contrast and cine MRI [21,22].

By demonstrating that a uniform, systematic one-voxel inward offset effectively mitigates severe boundary contamination for highly sensitive parameters while simultaneously preserving the stability of central metrics like kinetic energy, this study establishes a highly pragmatic framework for standardising image analysis. Achieving a singular, stable, and noise-reduced measurement pipeline is an essential prerequisite for deploying these advanced haemodynamic biomarkers in multi-centre clinical trials, ensuring analytical simplicity and longitudinal reproducibility when monitoring disease or assessing response to intervention.

### 4.4. Limitations

A notable limitation of this mechanistic proof-of-concept study is the small sample size, which limits broad clinical generalisations and introduces constraints on non-parametric post hoc testing, increasing the risk of Type II errors. To mitigate this, our analysis incorporated Hedges’ g effect sizes, which demonstrated the large magnitude of measurement reduction caused by boundary erosion in the gradient-based parameters. Furthermore, a post hoc power analysis demonstrated that despite the limited cohort, the study maintained moderate-to-excellent statistical power to detect these differences at a significance level of 0.05. Second, true in vivo ground truth for viscous energy loss and vorticity is difficult to establish, which limits any definitive statement regarding the most physiologically accurate contouring method. Concrete routes to establishing such a reference standard in future work include computational fluid dynamics modelling, flow phantoms with known ground-truth velocity fields, and complementary modalities such as echo-particle image velocimetry (echo-PIV). Accordingly, the present study should be interpreted as demonstrating the magnitude of measurement variation introduced by boundary placement and highlighting the critical need for methodological standardisation. We did not evaluate wall shear stress, which is not conventionally assessed within the cardiac chambers and whose reliable near-wall estimation at the present spatial resolution is itself dominated by the partial-volume and boundary-layer effects that are the subject of this study; characterising the influence of contour offset on wall shear stress, ideally at higher spatial resolution and extended to the thoracic aorta, is a logical extension of this work. In addition, the qualitative visual grading was performed by a single experienced operator and was intended as a supportive, illustrative corroboration of the operator-independent quantitative endpoints, upon which the conclusions do not rest; a formal multi-rater inter-observer reliability assessment would be a valuable addition to future work. Finally, the morphological erosion in this study was applied in a two-dimensional, slice-by-slice manner. A 2D erosion successfully clears boundary noise within the reconstructed axial plane but leaves the through-plane boundaries unaltered. While this 2D approach effectively removed the majority of artefactual interface noise to demonstrate the underlying mechanistic principles, future studies utilising a true 3D spherical morphological erosion will be required to ensure uniform boundary clearance across all spatial dimensions.

## 5. Conclusions

Systematic inward contour offsets significantly influence the quantification of highly sensitive four-dimensional flow cardiovascular magnetic resonance-derived haemodynamic parameters. For gradient-based measures such as vorticity and viscous energy loss, a systematic one-voxel offset effectively eliminates severe boundary-related noise while preserving the true physiological flow core. Because the change in kinetic energy under erosion reflects the loss of integration volume rather than boundary artefacts, extracting it from baseline contours remains physiologically sound. Nevertheless, to ensure methodological simplicity and strict standardisation across clinical analysis pipelines, we recommend the uniform application of a one-voxel offset for the comprehensive extraction of all advanced intracardiac haemodynamic parameters.

## Figures and Tables

**Table 1:** Cohort Characteristics and Baseline Volumetrics.

Parameter	Value (Mean ± SD)
Age (years)	76.6 ± 3.4
Sex (Male/Female)	3/2
Heart Rate (bpm)	72 ± 11
BSA (m^2^)	1.85 ± 0.28
LV End-Diastolic Volume (mL)	176.0 ± 76.5
LV End-Systolic Volume (mL)	65.3 ± 27.6
LV Ejection Fraction (%)	62.9 ± 5.5
NT-proBNP (pg/mL)	1084 ± 1572
eGFR (mL/min/1.73 m^2^)	71.0 ± 15.6

LV = left ventricle.

**Table 2:** Summary of Full-RR Haemodynamic Parameters.

Chamber	Metric	Baseline	1-Voxel Offset	2-Voxel Offset	Hedges’ g Baseline vs. 1-Voxel Offset	Hedges’ g 1-Voxel vs. 2-Voxel Offset
Left ventricle	Full-RR Vorticity (s^−1^)	249.6 ± 79.9	211.1 ± 61.0	157.0 ± 60.4	2.23	2.03
	Full-RR Energy Loss (µW)	549.4 ± 303.0	434.9 ± 253.0	351.3 ± 230.0	1.72	2.33
	Full-RR Kinetic Energy (mJ)	1.86 ± 1.13	1.64 ± 1.04	1.37 ± 0.94	—	—
Right ventricle	Full-RR Vorticity (s^−1^)	196.4 ± 31.6	156.6 ± 24.3	103.8 ± 15.0	7.03	6.47
	Full-RR Energy Loss (µW)	481.5 ± 173.1	279.6 ± 106.1	173.6 ± 71.9	1.57	1.89
	Full-RR Kinetic Energy (mJ)	1.28 ± 0.57	1.11 ± 0.53	0.87 ± 0.44	—	—
Left atrium	Full-RR Vorticity (s^−1^)	237.6 ± 105.4	190.4 ± 89.5	132.0 ± 73.2	2.92	2.70
	Full-RR Energy Loss (µW)	828.4 ± 505.2	426.9 ± 314.0	240.2 ± 180.4	1.65	1.11
	Full-RR Kinetic Energy (mJ)	1.12 ± 0.69	0.87 ± 0.57	0.66 ± 0.45	—	—
Right atrium	Full-RR Vorticity (s^−1^)	243.4 ± 87.5	197.3 ± 79.3	140.9 ± 63.1	6.47	4.42
	Full-RR Energy Loss (µW)	833.5 ± 223.8	429.1 ± 154.4	264.0 ± 132.2	3.27	5.03
	Full-RR Kinetic Energy (mJ)	1.29 ± 0.45	1.05 ± 0.41	0.84 ± 0.36	—	—

Data are presented as mean ± standard deviation. Phase-specific peak systolic, E-wave, and A-wave data are detailed in Supplementary Tables S1 and S2 and illustrated in Supplementary Figure S1.

## Data Availability

The original contributions presented in this study are included in the article/supplementary material. Further inquiries can be directed to the corresponding author(s).

## Supplementary Materials

The following supporting information can be downloaded at https://www.mdpi.com/article/10.3390/jcdd13060280/s1: Figure S1: Phase-specific retention of advanced haemodynamic parameters with progressive contour erosion. Kinetic energy, vorticity, and viscous energy loss for each cardiac chamber at peak systole, peak E-wave, and peak A-wave, expressed as a percentage of the baseline (unmodified-contour) value at one- and two-voxel inward offsets. Data are cohort means (n = 5). Corresponding full-cardiac-cycle data are shown in Figure 3, and the underlying values are tabulated in Supplementary Tables S1 and S2; Table S1: Phase-specific vorticity (s^−1^) across contouring methods; Table S2: Phase-specific viscous energy loss (μW) across contouring methods; Table S3: Qualitative visual grading of boundary interface noise across contouring methods.

## Abbreviations

The following abbreviations are used in this manuscript:

CMR	Cardiac magnetic resonance imaging
EDV	End diastolic volume
EF	Ejection fraction
ESV	End systolic volume
KE	Kinetic energy
LA	Left atrium
LV	Left ventricle
RA	Right atrium
RV	Right ventricle
vEL	Viscous energy loss
