# Supplementary material for: Impact of Contour Boundary Offsets on 4D Flow CMR-Derived Intracardiac Haemodynamic Parameters
Source: J Cardiovasc Dev Dis. 2026 Jun 22;13(6):280. doi: 10.3390/jcdd13060280 (PMC13302185; doi:10.3390/jcdd13060280)
Supplement: Supplementary file 1 [file jcdd-13-00280-s001.zip › jcdd-4329808-supplementary.pdf]

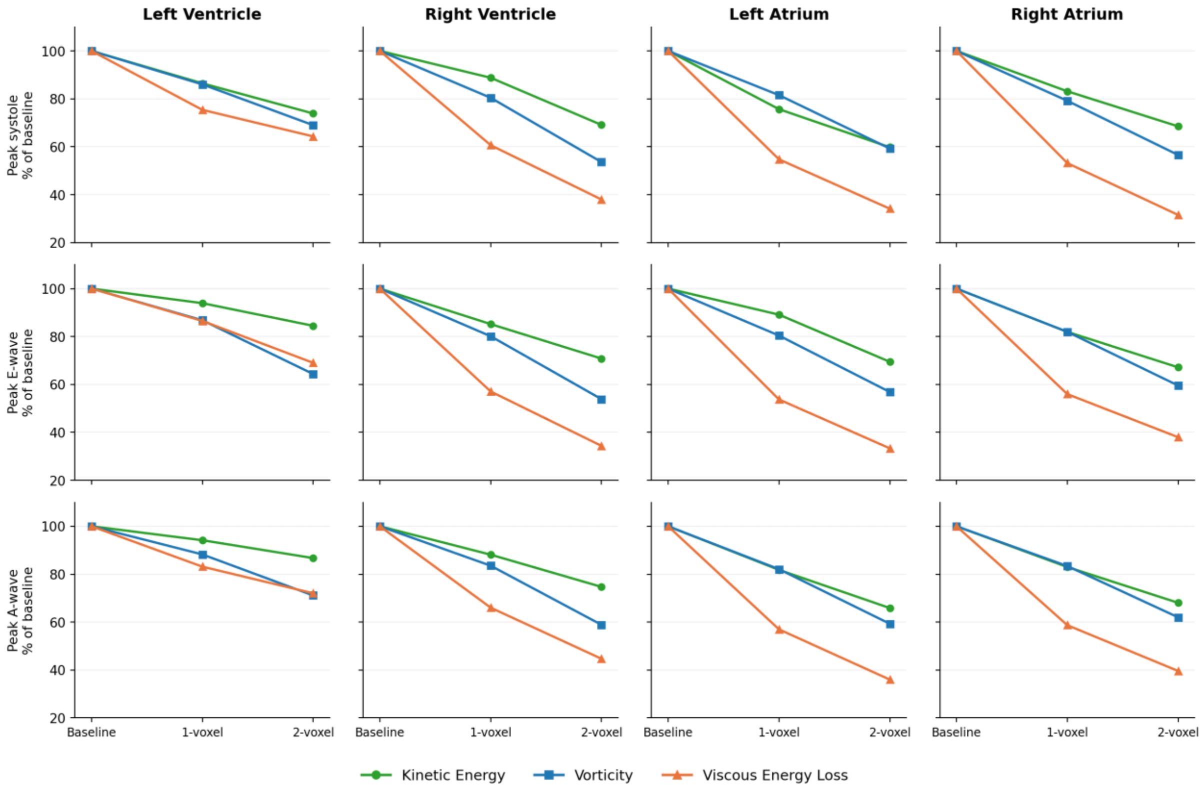

Supplementary Figure S1. Phase-specific retention of advanced haemodynamic parameters with progressive contour erosion. Kinetic energy, vorticity, and viscous energy loss for each cardiac chamber at peak systole, peak E-wave, and peak A-wave, expressed as a percentage of the baseline (unmodified-contour) value at one- and two-voxel inward offsets. Data are cohort means (n = 5). Corresponding full-cardiac-cycle data are shown in Figure 3, and the underlying values are tabulated in Supplementary Tables S1 and S2.

Table S1. Phase-specific vorticity (s^{-1}) across contouring methods.

Chamber	Cardiac phase	Baseline	1-voxel offset	2-voxel offset
Left ventricle	Peak systole	275.5	237.0	190.1
	Peak E-wave	399.2	346.4	256.8
	Peak A-wave	305.3	269.4	217.1
Right ventricle	Peak systole	277.0	222.8	148.4
	Peak E-wave	210.1	168.3	113.1
	Peak A-wave	234.5	195.9	138.0
Left atrium	Peak systole	268.1	218.6	158.8
	Peak E-wave	297.8	239.4	169.2
	Peak A-wave	294.5	241.7	174.6
Right atrium	Peak systole	277.1	219.4	156.7
	Peak E-wave	319.1	261.5	189.9
	Peak A-wave	264.9	220.9	164.2

Values are cohort mean (s^{-1}) ($n = 5$). Full-cardiac-cycle values for these parameters are reported in Table 2 of the main manuscript. LV, left ventricle; RV, right ventricle; LA, left atrium; RA, right atrium.

Table S2. Phase-specific viscous energy loss (µW) across contouring methods.

Chamber	Cardiac phase	Baseline	1-voxel offset	2-voxel offset
Left ventricle	Peak systole	979.0	738.1	629.5
	Peak E-wave	997.6	862.9	688.3
	Peak A-wave	1105.3	918.9	796.0
Right ventricle	Peak systole	1055.5	640.0	400.5
	Peak E-wave	442.0	252.1	151.7
	Peak A-wave	729.9	481.8	326.0
Left atrium	Peak systole	1089.7	596.4	371.6
	Peak E-wave	1259.2	676.2	419.2
	Peak A-wave	1346.3	766.8	484.3
Right atrium	Peak systole	1089.3	579.0	343.3
	Peak E-wave	1300.0	727.2	493.3
	Peak A-wave	1079.6	633.7	427.3

Values are cohort mean (µW) (n = 5). Full-cardiac-cycle values for these parameters are reported in Table 2 of the main manuscript. LV, left ventricle; RV, right ventricle; LA, left atrium; RA, right atrium.

Table S3. Qualitative visual grading of boundary interface noise across contouring methods.

Case	Vorticity			Viscous energy loss		
	Baseline	1-voxel	2-voxel	Baseline	1-voxel	2-voxel
1	1	3	3	0	2	3
2	1	2	3	0	2	2
3	2	2	3	0	2	2
4	2	3	3	1	3	3
5	2	3	3	0	2	2
**Average**	**1.6**	**2.6**	**3.0**	**0.2**	**2.2**	**2.4**

Boundary interface noise was graded on a 4-point ordinal scale by a single experienced operator: 0 = severe, 1 = moderate, 2 = mild, 3 = clean. Baseline, 1-voxel and 2-voxel denote the contouring method.
